# M-mode echocardiographic reference values in Pantja goats

**DOI:** 10.14202/vetworld.2017.22-28

**Published:** 2017-01-10

**Authors:** Parul Singh, Narendra Singh Jadon, Deepti Bodh, Manjul Kandpal

**Affiliations:** Department of Veterinary Surgery & Radiology, College of Veterinary & Animal Sciences, GBPUAT, Pantnagar - 263 145, Uttarakhand, India

**Keywords:** echocardiography, goats, heart disease, M-mode, Pantja

## Abstract

**Aim:**

The aim of this study was to establish M-mode echocardiographic reference values in Pantja goats and to study the effect of gender and body weight (BW) on these parameters.

**Materials and Methods:**

A total of 18, clinically healthy, adult Pantja goats of either sex, aged 2-4 years and weighing 10-44 kg were included in the study. Echocardiographic examination was performed in the standing unsedated animal. All measurements were made from the right parasternal long-axis left ventricular outflow tract view of the heart. The following parameters were recorded: Left ventricular internal diameter at diastole and systole, interventricular septal thickness at diastole and systole, left ventricular posterior wall (LVPW) thickness at diastole and systole, end diastolic and systolic volumes, stroke volume, fractional shortening, ejection fraction, percent systolic thickening of interventricular septum, percent systolic thickening of LVPW, cardiac output, left atrial (LA) diameter at diastole and systole, aortic (AO) root diameter at diastole and systole, LA/AO, LA posterior wall thickness at diastole and systole, left ventricular ejection time, DE amplitude, EF slope, AC interval and e-point to septal separation.

**Results:**

This study demonstrated specific reference ranges of M-mode echocardiographic parameters and indices in healthy Pantja goats. Normal echocardiographic values obtained in Pantja goats were quite different from other goat breeds. Gender had no influence on echocardiographic parameters, while high correlations were found between most echocardiographic parameters and BW.

**Conclusion:**

The echocardiographic values obtained in the study may serve as a reference for future studies in this breed, for cardiovascular disease diagnosis and for utilizing the goat as a model for cardiac disorders in humans.

## Introduction

Echocardiography is a non-invasive method for assessment of cardiac structure and function. In goats, structural cardiac abnormalities are rarely diagnosed clinically because this species is relatively resistant to cardiac disease and is rarely presented for detailed medical evaluation at veterinary hospitals. Despite this, echocardiography has proved to be an interesting tool for diagnosing cardiovascular diseases in goats [1,2].

Unlike other animals, goats are easily manageable and have a heart size comparable with that of human beings, which makes them an ideal candidate for the developing of animal models for human cardiovascular research [3]. However, for accurate measurement of cardiac dimensions and to evaluate possible changes in the cardiac function, reference values are required. With the exception of a few goat breeds [4-8], published information on the full set of reference ranges for cardiac dimensions and indices for Indian goats is lacking in literature.

Pantja is the local goat of Tarai region of Uttarakhand. Studies on body weight (BW), body measurements, scrotal morphology, production traits, growth pattern, and mortality rate of Pantja goats [9-11] have been reported in literature. There are no reports available on the two-dimensional and M-mode echocardiographic study in Pantja goats. This study was designed to establish reference values of two-dimensional guided M-mode echocardiographic parameters and indices in healthy Pantja goats and to evaluate the possible effect of gender and BW on these parameters.

## Materials and Methods

### Ethical approval

This study was conducted in accordance with the guidelines laid down by the Institutional Animal Ethics Committee.

### Animals

A total of 18, clinically healthy, adult Pantja goats (9 males, 9 females), aged 2-4 years and weighing 10-44 kg (males=29.22±8.44 kg, females=22.88±5.32 kg, mean=26.61±2.52 kg) were included in the study. Animals were acclimatized to approaching, handling and housing conditions for a period of 21-28 days before start of the echocardiographic examination. Animals were considered to be free of cardiovascular disease based on history, clinical examination, complete hematobiochemical examination, cardiac auscultation, normal lead II electrocardiogram, and routine echocardiography.

### Echocardiographic protocol

Echocardiographic examination was carried out with a Scintilla Color Doppler Ultrasound Scanner (Larsen and Toubro Limited) equipped with 2.5-3.5 MHz multi-frequency phased-array transducer. Region between 3^rd^ to 5^th^ right intercostal spaces starting just caudal to the triceps muscle mass, from 3 to 5 cm below right olecranon to 5-10 cm above it was prepared for echocardiographic examination. Echocardiography was performed in a standing position with the right front leg pulled slightly forward by an assistant. M-mode echocardiographic evaluation of heart was guided by simultaneous display of real time two-dimensional echocardiographic images.

### Measurements

**Figure-1:**
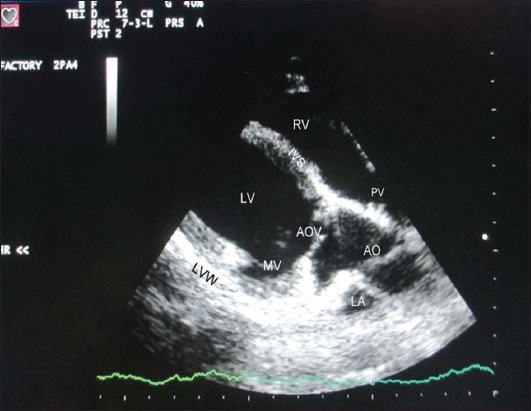
Echocardiogram showing right parasternal long axis left ventricular outflow view of heart. RV=Right ventricle, PV=Pulmonary valve, IVS=Interventricular septum, LA=Left atrium, LV=Left ventricle, MV=Mitral valve, AO=Aorta, AOV=Aortic valve, LVW=Left ventricular wall.

**Figure-2:**
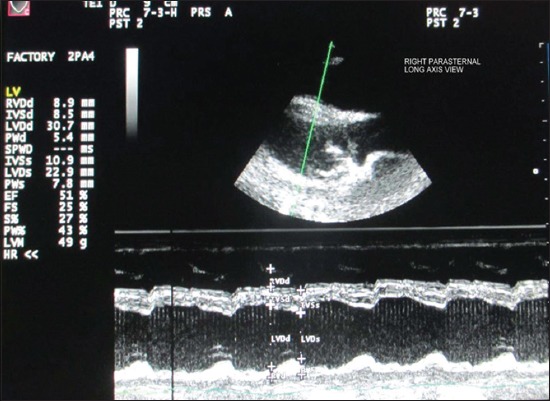
Echocardiogram showing simultaneous display of B-mode and M-mode images of left ventricle. LVDd=Left ventricular internal diameter at diastole, LVDs=Left ventricular internal diameter at systole, IVSd=Interventricular septum thickness at diastole, IVSs=Interventricular septum thickness at systole, LVPWd=Left ventricular posterior wall thickness at diastole, LVPWs=Left ventricular posterior wall thickness at systole.

**Figure-3:**
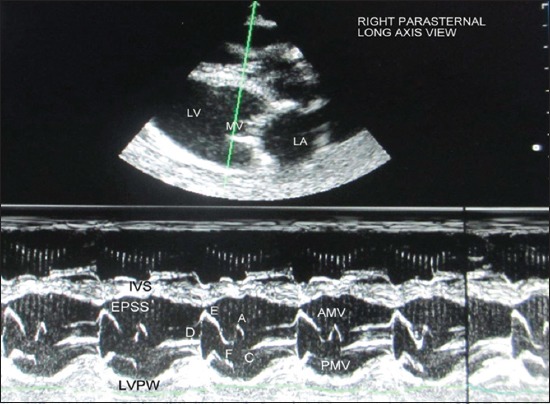
M-mode echocardiogram showing characteristic motion of anterior MV leaflet. D, point of MV opening; E, maximum MV opening during rapid ventricular filling; F, end of rapid ventricular filling; A, peak MV motion secondary to atrial contraction; C, point of MV closure; EPSS=Distance between E point to interventricular septum, LV=Left ventricle, MV=Mitral valve, LA=Left atrium, IVS=Interventricular septum, LVPW=Left ventricular posterior wall.

**Figure-4:**
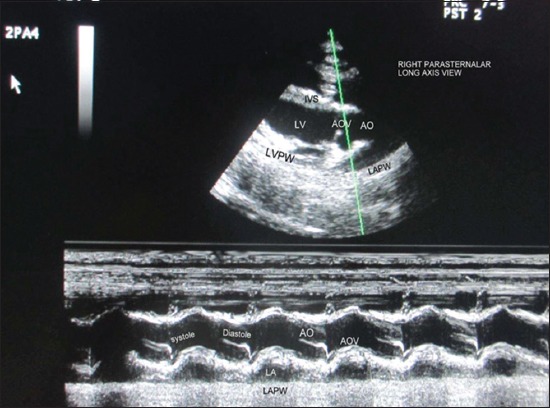
Echocardiogram showing M-mode measurement of left atrium and aorta in the right parasternal long axis view of heart; AO=Aorta, AOV=Aortic valve, LA=Left atrium, LAPW=Left atrium posterior wall, LV=Left ventricle, IVS=Interventricular septum, LVPW=Left ventricular posterior wall.

Echocardiographic examination was performed using leading edge method as per the recommendations of the American Society of Echocardiography from frozen images on the screen [12]. All measurements were made from right parasternal long-axis left ventricular outflow tract view of heart (Figure-1). For obtaining left ventricular images, cursor was positioned at the level just posterior to the chordae tendinae, perpendicular to the interventricular septum (IVS), and left ventricular posterior wall (LVPW) (Figure-2). The following parameters were recorded: Left ventricular internal diameter at diastole (LVDd) and systole (LVDs), IVS thickness at diastole (IVSd) and IVS thickness at systole (IVSs), LVPW thickness at diastole (LVPWd) and LVPW thickness systole (LVPWs). With the cursor line positioned perpendicularly over the mitral valves (MVs), two leaflets produced an “M” shaped image in inverse fashion on M-mode (Figure-3). Various points of this M-mode MV images were identified and following measurements were recorded: DE amplitude (MV excursion amplitude), EF slope (early diastolic posterior motion of MVs), AC interval (time interval between peak of A-wave and C point), and e-point to septal separation (EPSS). Slight pointing of the transducer further anteriorly and toward the spine brought aorta and left atrium into view (Figure-4). The following measurements were recorded: Left atrial (LA) diameter at diastole (LAd) and LA diameter at systole (LAs), aortic diameter at diastole (AOd) and AO diameter at systole (AOs), LA/AO, LA posterior wall thickness at diastole (LAPWd) and LA posterior wall thickness at systole (LAPWs) and left ventricular ejection time (LVET).

Teicholz formula [13] was used to calculate the end diastolic volume and end systolic volume (EDV and ESV, respectively). Other parameters, viz., fractional shortening (FS), ejection fraction (EF), stroke volume (SV), cardiac output (CO), percent systolic thickening of IVS%, and percent systolic thickening of LVPW% were calculated using established formulae suggested by Kienle [14]. The animals were grouped into six classes based on their BW: Group I (10-16 kg), Group II (16-22 kg), Group III (22-28 kg), Group IV (28-34 kg), Group V (34-40 kg), and Group VI (40-44 kg) to study the effect of BW on various echocardiographic parameters.

### Statistical analysis

Data were analyzed using statistical software SPSS 14.0. Independent sample t-test was used to study differences between male and female dogs. Mean values among different groups based on BW was compared using one-way ANOVA and Duncan’s multiple range test as per methods described by Snedecor and Cochran [15]. Linear regression analysis was done to assess the relationship between echocardiographic parameters and BW. The correlation was considered positive and significant when correlation coefficient was >0.40 and significance was <0.01. Values of p<0.01 was considered significant for all statistical tests.

## Results and Discussion

M-mode echocardiographic reference values in Pantja goats were determined and its subjective comparison with other goat breeds was done. In addition, the possible effect of gender and BW on echocardiographic parameters was determined. Mean±standard error of M-mode echocardiographic parameters and indices in Pantja goats is presented in Table-1. Data on the basis of gender and BW are presented in Tables-1 and 2, respectively. Regression equations, coefficient of determination, and correlation coefficients of various echocardiographic parameters, when compared with BW, are presented in Table-3. Subjective comparison of echocardiographic parameters in Pantja goats with published values in various breed of goats is presented in Table-4.

**Table-1:** Mean±SE of M-mode echocardiographic parameters and indices in Pantja goats.

Parameters	Mean±SE	Male	Female	Reference range
LVDd (mm)	29.54±1.29	30.73±2.49	28.59±1.25	19.5-40.1
LVDs (mm)	19.20±1.19	20.22±2.40	18.39±1.02	10.1-26.7
IVSd (mm)	8.32±0.26	8.15±0.43	8.46±0.34	6.1-10
IVSs (mm)	11.16±0.39	10.7±0.48	11.53±0.58	8.5-13.7
LVPWd (mm)	7.41±0.42	7.71±0.80	7.18±0.45	3.9-10.5
LVPWs (mm)	10.36±0.63	11.2±1.21	9.69±0.59	6.1-17.1
EDV (ml)	35.78±3.69	39.90±7.19	32.47±3.40	11.93-70.41
ESV (ml)	12.86±1.86	15.43±3.71	10.8±1.47	21.1-30.07
SV (ml)	22.91±2.20	24.28±4.35	21.66±2.07	9.81-45.95
CO (ml/min)	1958.71±207.72	2133.89±400.12	1803±193.56	785.36-3860.05
FS (%)	35.70±1.90	35.50±3.92	35.86±1.67	21.7-48.20
EF (%)	66.30±2.50	64.93±5.29	67.4±1.89	44.99-82.28
IVS (%)	31.82±2.56	32.35±4.63	31.39±2.98	12.19-55.73
LVPW (%)	39.51±3.46	46.29±5.29	34.09±4.00	14.49-62.85
LAd (mm)	21.76±0.83	22.18±1.35	21.43±1.10	16.7-30.4
LAs (mm)	24.36±1.00	24.8±1.64	24.02±1.31	18.6-34.9
AOd (mm)	20.39±0.81	20.96±1.24	19.94±1.11	16.3-29.1
AOs (mm)	22.72±0.86	23.37±1.30	22.21±1.18	18.6-31
LA/AO	1.06±0.03	1.04±0.04	1.08±0.04	0.81-1.192
LAPWd (mm)	11.04±0.51	12.35±0.60	10±0.61	7-14.7
LAPWs (mm)	9.15±0.51	10.28±0.57	8.24±0.68	6.2-13.6
LVET (ms)	228.88±6.79	230.5±10.41	227.86±9.44	184-273
DE amplitude (mm)	11.72±0.76	12.42±1.1	11.16±1.01	6.7-17.6
EF slope (mm/s)	122.44±4.49	127.5±5.90	118.4±6.55	70-140
AC interval (ms)	92.55±4.15	89.5±4.98	95±6.45	64-146
EPSS (mm)	4.35±0.26	4.4±0.49	4.32±0.29	2.1-5.9

LVDd=Left ventricular internal diameter at diastole, LVDs=Left ventricular internal diameter at systole, IVSd=Interventricular septum thickness at diastole, IVSs=Interventricular septum thickness at systole, LVPWd=Left ventricular posterior wall thickness at diastole, LVPWs=Left ventricular posterior wall thickness at systole, EDV=End diastolic volume, ESV=End systolic volume, SV=Stroke volume, CO=Cardiac output, FS=Fractional shortening, EF=Ejection fraction, IVS=Interventricular septum, LVPW=Left ventricular posterior wall, LAd=Left atrial diameter at diastole, LAs=Left atrial diameter at systole, AOd=Aortic diameter at diastole, AOs=aortic diameter at systole, LA/AO=Left atrial to aortic ratio, LAPWd=Left atrial posterior wall thickness at diastole, LAPWs=Left atrial posterior wall thickness at systole, LVET=Left ventricular ejection time, EPSS=E-point to septal separation, SE=Standard error

**Table-2:** Mean±SE of M-mode echocardiographic parameters and indices on the basis of body weight.

Body weight (kg)						

Parameters	I (10-16 kg)	II (16-22 kg)	III (22-28 kg)	IV (28-34 kg)	V (34-40 kg)	VI (40-44 kg)
LVDd (mm)	22.7±2.07^a^	25.2±0.55^a^	27.6±1.09^a^	30.73±0.56^b^	33.03±0.56^b^	38.00±1.24^c^
LVDs (mm)	13.46±2.12^a^	15.4±1.32^a^	17.03±2.11^a^	21.00±1.35^ab^	23.46±1.93^a^	24.86±1.78^b^
IVSd (mm)	6.46±0.18^a^	8.06±0.75^a^	8.36±0.39^a^	9.06±0.18^b^	9.03±0.52^ab^	9.93±0.27^c^
IVSs (mm)	8.96±0.29^a^	11.43±1.48^b^	11.43±0.44^b^	12.03±0.38^c^	11.26±1.31^b^	11.83±0.57^bc^
LVPWd (mm)	4.63±0.54^a^	7.46±1.09^b^	6.96±0.17^b^	7.93±0.48^bc^	8.2±0.66^bc^	9.3±0.96^c^
LVPWs (mm)	6.56±0.32^a^	10.46±0.85^b^	9.16±0.72^ab^	10.6±0.35^b^	11.13±0.40^c^	14.23±1.80^d^
EDV (ml)	18.05±4.17^a^	22.80±1.23^a^	30.26±2.89^b^	37.16±0.16^b^	44.28±1.83^c^	62.15±4.82^d^
ESV (ml)	5.03±2.01^a^	6.67±1.53^a^	8.92±2.47^ab^	14.62±2.45^b^	19.51±3.91^b^	22.41±4.03^c^
SV (ml/beat)	13.02±2.15	16.12±1.38	21.30±0.80	22.54±1.07	24.76±3.0	39.73±3.77
CO (ml/min)	1306.24±52.81^a^	1306.24±52.81^a^	1871.73±95.50^b^	1883.76±174.64^b^	2033.57±249.8^c^	3507.75±338.7^c^
FS (%)	41.38±3.93^a^	38.93±4.63^a^	38.52±6.46^a^	31.77±3.21^a^	29.04±5.17^a^	34.59±3.76^a^
EF (%)	73.94±4.76^a^	70.91±5.76^a^	71.60±6.18^a^	61.03±4.80^ab^	56.24±7.60^b^	64.09±4.94^ab^
IVS (%)	36.41±9.73^a^	40.88±5.92^a^	36.83±3.26^a^	26.04±3.14^b^	20.55±4.37^b^	30.20±4.45^a^
LVPW (%)	43.85±9.03^a^	38.38±14.44^a^	31.42±8.59^b^	34.05±3.66^b^	36.93±7.98^b^	52.38±5.23^c^
LAd (mm)	19.93±1.14^a^	18.7±1.44^a^	21.2±0.32^ab^	21.03±0.26^ab^	22.4±0.55^ab^	27.33±3.06^c^
LAs (mm)	22.43±1.47^a^	21.63±1.82^a^	22.76±0.89^a^	23.03±0.96^a^	25.26±0.71^a^	31.06±3.63^b^
AOd (mm)	18.5±0.68^a^	19.70±1.10^a^	18.76±0.38^a^	17.63±0.81^a^	21.93±1.85^b^	25.83±2.17^b^
AOs (mm)	20.93±1.24^a^	21.66±1.98^a^	21.43±0.93^a^	19.53±0.58^a^	23.86±1.23^ab^	28.93±1.34^b^
LA/AO	1.08±0.09	0.95±7.08	1.13±0.03	1.19±0.03	0.97±0.08	1.05±0.04
LAPWd (mm)	9.83±0.38^a^	9.9±0.32^a^	9.96±1.58^a^	11.53±2.10^b^	11.63±0.49^b^	13.4±1.10^c^
LAPWs (mm)	8.4±0.25^a^	7.5±0.45^a^	7.9±0.92^a^	8.7±1.64^a^	9.5±0.32^b^	12.9±0.36^c^
LVET (ms)	200.66±5.48	210±19.42	220.66±5.60	248.33±9.61	243.66±23.58	250±11.76
DE amp. (mm)	7.76±0.78^a^	11.2±1.87^b^	12.33±0.62^b^	9.4±1.13^c^	13.6±1.15^b^	16.03±0.95^bc^
EF slope (mm/s)	123.33±8.81^a^	113.33±8.81^a^	133.33±3.33^a^	100±17.32^b^	130±10.00^b^	134.66±2.90^b^
AC int (ms)	74.33±5.54^a^	103.66±21.9^b^	87.00±4.00^a^	94.66±9.40^b^	97.33±2.33^b^	98.33±2.02^b^
EPSS (mm)	2.8±0.60^a^	4.36±0.77^b^	4.3±0.11^b^	4.86±0.49^bc^	4.46±0.73b^c^	5.33±0.18^c^

LVDd=Left ventricular internal diameter at diastole, LVDs=Left ventricular internal diameter at systole, IVSd=Interventricular septum thickness at diastole, IVSs=Interventricular septum thickness at systole, LVPWd=Left ventricular posterior wall thickness at diastole, LVPWs=Left ventricular posterior wall thickness at systole, EDV=End diastolic volume, ESV=End systolic volume, SV=Stroke volume, CO=Cardiac output, FS=Fractional shortening, EF=Ejection fraction, IVS=Interventricular septum, LVPW=Left ventricular posterior wall, LAd=Left atrial diameter at diastole, LAs=Left atrial diameter at systole, AOd=Aortic diameter at diastole, AOs=aortic diameter at systole, LA/AO=Left atrial to aortic ratio, LAPWd=Left atrial posterior wall thickness at diastole, LAPWs=Left atrial posterior wall thickness at systole, LVET=Left ventricular ejection time, EPSS=E-point to septal separation, SE=Standard error.

a, b, c, d Values with different superscript differ significantly (p<0.01) between groups. Values with same superscript did not differ significantly (p<0.01) between groups

**Table-3:** Regression equations, coefficient of determination and correlation coefficients of various echocardiographic parameters when compared to body weight in Pantja goats.

Parameters	Regression (y)	Coefficient of determination (r^2^)	Correlation coefficient (r)
LVDd (mm)	y=0.486x+16.598	0.894	0.946**
LVDs (mm)	y=0.403x+0.894	0.723	0.850**
IVSd (mm)	y=0.074x+6.350	0.495	0.703**
LVPWd (mm)	y=0.127x+4.031	0.560	0.748**
LVPWs (mm)	y=0.200x+5.049	0.622	0.789**
EDV (ml)	y=1.374x−0.77	0.872	0.934**
ESV (ml)	y=0.622x−3.689	0.704	0.839**
SV (ml/beat)	y=0.751x+2.948	0.734	0.857**
CO (ml/min)	y=63.122x+127.73	0.534	0.731**
LAd (mm)	y=0.216x+16.015	0.421	0.649**
LAs (mm)	y=0.248x+17.759	0.385	0.621**
AOd (mm)	y=0.188x+15.393	0.337	0.580**
AOs (mm)	y=0.201x+17.391	0.341	0.584**
LAPWs (mm)	y=0.142x+5.363	0.481	0.694**
DE amplitude (mm)	y=0.201x+17.391	0.473	0.688**
EPSS (mm)	y=0.069x+2.519	0.424	0.651**

y=Achieved value of echocardiographic parameter at body weight x.

** Denotes the significance level of 1%. LVDd=Left ventricular internal diameter at diastole, LVDs=Left ventricular internal diameter at systole, IVSd=Interventricular septum thickness at diastole, IVSs=Interventricular septum thickness at systole, LVPWd=Left ventricular posterior wall thickness at diastole, LVPWs=Left ventricular posterior wall thickness at systole, EDV=End diastolic volume, ESV=End systolic volume, SV=Stroke volume, CO=Cardiac output, FS=Fractional shortening, EF=Ejection fraction, IVS=Interventricular septum, LVPW=Left ventricular posterior wall, LAd=Left atrial diameter at diastole, LAs=Left atrial diameter at systole, AOd=Aortic diameter at diastole, AOs=aortic diameter at systole, LAPWs=Left atrial posterior wall thickness at systole, EPSS=E-point to septal separation

**Table-4:** Subjective comparison of M-mode echocardiographic parameters in Pantja goats with previously reported values in various breeds of goats [5-7].

Parameter	Pantja goats - Current study	Adult goats - Hallowell *et al.* [5]	Saanen goats - Leroux *et al*. [6]	Swedish goats - Olsson *et al*. [7]
LVDd (mm)	29.54±1.29	37.4±0.78	48.1±0.37	40.6±0.1
LVDs (mm)	19.20±1.19	21.1±0.31	27.4±0.24	24.0±0.8
IVSd (mm)	8.32±0.26	9.8±0.21	8.8±0.77	NR
IVSs (mm)	11.16±0.39	13.2±0.32	14.8±0.11	NR
LVPWd (mm)	7.41±0.42	7.9±0.07	9.4±0.09	6.8±0.03
LVPWs (mm)	10.36±0.63	12.2±0.19	15.3±0.09	12.9±0.06
EDV (ml)	35.78±3.69	NR	109.16±19.40	NR
ESV (ml)	12.86±1.86	NR	28.42±6.10	NR
SV (ml/beat)	22.91±2.20	NR	80.73±15.17	NR
FS (%)	35.70±1.90	45.2±5.9	43.0±3.11	40.6±7.9
EF (%)	66.30±2.50	73.6±5.4	73.9±3.58	NR
IVS (%)	31.82±2.56	NR	68.81±10.08	NR
LVPW (%)	39.51±3.46	NR	65.54±13.7	NR
LAd (mm)	21.76±0.83	NR	41.5±0.22	26.9±0.3
LAs (mm)	24.36±1.00	40.6±0.54	47.6±0.22	NR
AOd (mm)	20.39±0.81	26.4±0.30	28.3±0.10	NR
AOs (mm)	22.72±0.86	NR	NR	NR
LVET (ms)	228.88±6.79	180±0.05	NR	NR
DE amplitude (mm)	11.72±0.76	11.72±0.76	NR	NR
EF slope (mm/s)	122.44±4.49	NR	NR	NR
AC interval (ms)	92.55±4.15	NR	NR	NR
EPSS (mm)	4.35±0.26	NR	NR	NR
EF slope (mm/s)	122.44±4.49	NR	NR	NR
AC interval (ms)	92.55±4.15	NR	NR	NR
EPSS (mm)	4.35±0.26	3.7±0.09	NR	NR

LVDd=Left ventricular internal diameter at diastole, LVDs=Left ventricular internal diameter at systole, IVSd=Interventricular septum thickness at diastole, IVSs=Interventricular septum thickness at systole, LVPWd=Left ventricular posterior wall thickness at diastole, LVPWs=Left ventricular posterior wall thickness at systole, EDV=End diastolic volume, ESV=End systolic volume, SV=Stroke volume, CO=Cardiac output, FS=Fractional shortening, EF=Ejection fraction, IVS=Interventricular septum, LVPW=Left ventricular posterior wall, LAd=Left atrial diameter at diastole, LAs=Left atrial diameter at systole, AOd=Aortic diameter at diastole, AOs=aortic diameter at systole, LVET=Left ventricular ejection time, EPSS=E-point to septal separation, SE=Standard error, NR: Not reported

LVDd and LVDs in Pantja goats was lower than adult goats, Saanen goats, and Swedish goats [5-7]. IVSd and IVSs was lower than adult goats and Saanen goats [5,6]. LVPWd and LVPWs in Pantja goats was lower than adult and Saanen goats [5,6] but greater than Swedish goats [7]. Left ventricular volumes, viz., EDV, ESV and SV in Pantja goats were lower than Saanen goats [6]. CO in Pantja goats was lower than values reported in Swedish goats during pregnancy (6730±0.72 ml/min), lactation (6120±0.52 ml/min), and dry period (4390±0.27 ml/min) [7]. FS and EF in Pantja goats was lower than adult goats and Saanen goats [5,6]. IVS% and LVPW% during ventricular contraction in Pantja goats was lower than Saanen goats [6]. LAd in Pantja goats was lower than Saanen goats and Swedish goats [6,7], whereas LAs and AOd in Pantja goats was lower than adult goats and Saanen goats [5,6]. LVET in Pantja goats was greater than adult goats [5]. LA/AO, LAPWd and LAPWs in Pantja goats were 1.06±0.03, 11.04±0.51 mm and 9.15±0.51 mm, respectively. DE amplitude, EF slope, and AC interval in Pantja goats was 11.72±0.76 mm (6.7-17.6 mm), 122.44±4.49 mm/s (70-140 mm/s) and 92.55±4.15 ms (64-146 ms), respectively. Decreased value of DE amplitude is suggestive of advanced cardiomyopathy [16] while alteration in the normal value of E-F slope denotes altered flow across the MVs [17]. AC interval represented the time interval between maximum opening of MV cusps during atrial systole and their coaptation at the end of atrial systole. Value of EPSS was 4.35±0.26 mm (2.1-5.9 mm). An increased EPSS is suggestive of enlarged left ventricle and dilated cardiomyopathy in Labrador retrievers [18]. Values of LA/AO, LAPWd, LAPWs, DE amplitude, EF slope, AC interval and EPSS were not determined in the previous studies [5-7].

Nonsignificant influence of gender on various echocardiographic parameters (LVDd, LVDs, IVSd, IVSs, LVPWd, LVPWs, EDV, ESV, SV, FS, EF, IVS%, LVPW%, LAs, AOd, LAPWd, LAPWs, LA/AO, DE amplitude and EPSS) in Pantja goats (Table-1) was in accordance with findings reported in Spitz, Labrador retriever and non-descript dogs [16]. On contrary, significant differences in several echocardiographic parameters were observed between male and female Philippine native sheep [19]. EDV, ESV, SV and CO in male Pantja goats was slightly higher than females, although the differences were non-significant (Table-1). Insignificant variation in heart rate with respect to gender of animals could have led to non-significant differences in the values among male and female Pantja goats. LA and AO diameter and LA/AO in Pantja goats (Table-1) was unaffected by gender. Similar findings were reported in Spitz, Labrador retriever and non-descript dogs [16]. Significantly (p<0.01) higher DE amplitude in male Pantja goats (Table-1) was in accordance with findings in Spitz dogs [20]. The reason for this could be higher average BW of males compared to females used in above studies. Non-significant effect of gender on values of EF slope, AC interval, and EPSS was in accordance with findings in Spitz, Labrador retriever and non-descript dogs [16]. Males had slightly higher EPSS which might be due to greater left ventricular dimensions in male Pantja goats compared to females.

BW correlated significantly (p<0.01) with most M-mode echocardiographic parameters (LVDd, LVDs, IVSd, LVPWd, LVPWs, EDV, ESV, SV, CO, LAs, AOd, LAPWs, DE amplitude, and EPSS) in Pantja goats (Tables-2 and 3). Similar findings were observed in Philippine sheep and sedated healthy growing female sheep [19,21]. It is seen that as BW of animal increases, the heart size also increases, and this might have contributed to the above findings. Significant (p<0.01) positive correlation between BW and IVSd, LVPWd and LVPWs in Pantja goats (Tables-2 and 3) was in accordance with findings in sedated healthy growing female sheep [21]. Left ventricular volumes, viz., EDV, ESV, SV, and CO showed significant (p<0.01) correlation with BW (Tables-2 and 3). This might be explained by the fact that with increase in BW, cardiac size increases and with increase in cardiac size, cardiac volume and CO also increases. A weak negative correlation of FS and EF with BW in Pantja goats was in accordance with previous findings in sheep [21]. Variation in the observation of these parameters can be explained by the fact that FS and EF are influenced by many parameters which include preload, afterload and contractility, all of which may act independently or in combination to affect this parameter [16]. Non-significant decrease in FS and EF values with increase in BW (Table-2) could also be due to the reduction of the fractional contraction which occurs as a result of BW increase in animals [16]. LADs, LADd, AOd, AOs and LAPWs correlated significantly (p<0.01) with BW in Pantja goats (Tables-2 and 3). Similar findings were reported in sedated healthy growing female sheep [21]. Among MV parameters, only DE amplitude showed significant (p<0.01) correlation with BW in Pantja goats (Tables-2 and 3). The increase in DE amplitude with an increase in BW can be explained by the rapid increase of left ventricular diameter with animal’s increasing BW and age. Similar findings were reported in Spitz and Labrador retriever dogs [16]. EPSS shows a very weak correlation to BSA and weight in some studies and no correlation in others; therefore, body size is generally not considered when assessing normal EPSS values [22]. However, significant (p<0.01) positive correlation between EPSS and BW in our study (Tables-2 and 3) could either be due to BW differences or small sample size. Similar findings were reported in sedated healthy growing female sheep [21].

## Conclusion

From the results of this study, it can be concluded that echocardiographic values in Pantja goats differ from other goat breeds. Gender had no influence while most echocardiographic parameters were influenced significantly by animal’s BW. Values obtained in the study may serve as a reference for future studies in this breed as well as for cardiovascular disease diagnosis in humans.

## Authors’ Contributions

PS: Research was done by this author as the part of her M.V.Sc. thesis dissertation. NSJ: Designed the study and supervised the research. DB, MK and PS: Analysed and interpreted the data. All authors read and approved the final manuscript.
